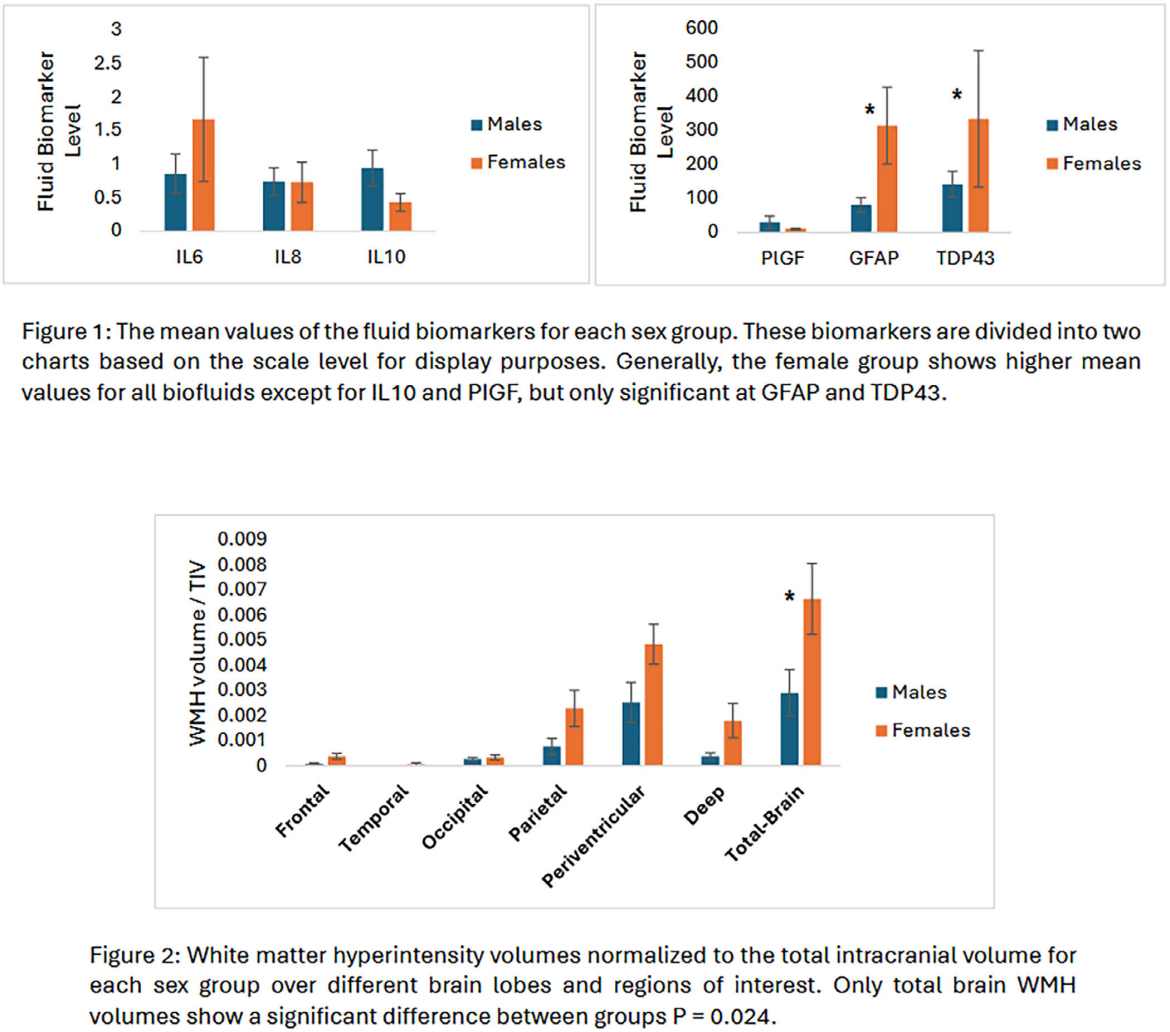# The Effect of Sex‐Differences on the Relationship Between White Matter Hyperintensity, Cerebrovascular Reactivity, and Fluid Biomarkers

**DOI:** 10.1002/alz.092095

**Published:** 2025-01-09

**Authors:** Ahmed A Bahrani, Yang Jiang, David K Powell, Yuriko Katsumata, Azadeh Nahvi, Tiffany Lee, Brian T Gold, Larry B Goldstein, Donna M. Wilcock, Gregory A Jicha, Peter T Nelson, Christopher M. Norris

**Affiliations:** ^1^ Sanders‐Brown Center on Aging, Lexington, KY USA; ^2^ University of Kentucky College of Medicine, Lexington, KY USA; ^3^ University of Kentucky Sanders‐Brown Center on Aging, Lexington, KY USA; ^4^ University of Kentucky, Lexington, KY USA; ^5^ College of Public Health, University of Kentucky, Lexington, KY USA; ^6^ Sanders‐Brown Center on Aging, University of Kentucky, Lexington, KY USA; ^7^ Indiana University School of Medicine, Stark Neurosciences Research Institute, Department of Neurology, Indianapolis, IN USA; ^8^ College of Medicine, University of Kentucky, Lexington, KY USA; ^9^ University of Kentucky College of Medicine, Sanders‐Brown Center on Aging, Lexington, KY USA

## Abstract

**Background:**

Alzheimer’s disease (AD) and vascular cognitive impairment and dementia (VCID) are the predominant types of dementia in older adults, associated with memory loss and cognitive deficits. White matter hyperintensities (WMH) are linked to both AD and VCID. Astrocytes play a crucial role in WM integrity, encompassing functions like neuroinflammation, oxidative stress, and Aβ clearance. Poorly reactive astrocytes could lead to implications, like WMH or vascular damage. This study aims to explore sex‐differences effect on the correlation between fluid biomarkers, WMH, and cerebrovascular reactivity (CVR).

**Method:**

Twenty‐seven participants (mean age 76.8±6.4 years, Female=15) preliminary data were collected from UK‐ADRC/MarkVCID cohorts. A correlation test was employed to examine sex‐differences based on the correlation of fluid inflammatory (GFAP, IL6, IL8, IL10), angiogenic (TDP‐43, and PlGF) biomarkers, and Aβ40 and 42, to global and regional CVR and WMH.

**Results:**

We observed several sex‐differences: the female group showed a significant correlation between WMH at occipital lobe and IL6 (P=0.031), IL10 (P=0.036), and GFAP (P=0.037), while male group only showed a significant correlation between Aβ42 and WMH at the occipital lobe (P=0.039). CVR data of the female group exhibited a correlation at the parietal lobe (right‐hemisphere) and IL8 (P=0.037) and Aβ40 (P=0.038) and between Aβ40 and CVR temporal lobe (right‐hemisphere, P=0.021). The male group showed a significant correlation between IL6 and CVR at the occipital lobe (left‐hemisphere, P=0.012. Generally, the female group showed higher mean values for all biomarkers except for IL10 and PIGF, but only significant at GFAP and TDP43. Additionally, the correlation test adjusted for age and sex showed that TDP‐43 had a significant correlation with WMH in the temporal (P=0.041), occipital (P=0.024), and parietal (P=0.024) lobes, while GFAP displayed a significant correlation only with WMH in the frontal lobe (P=0.013).

**Conclusions:**

Despite the small sample size, which warrants expansion in future studies, we observed interesting findings of sex‐differences in specific brain regions in relation to fluid biomarkers. These biomarkers may arise, in part, from reactive astrocytes, commonly found near many brain lesions, including WM pathology. Further studies are needed to gain deeper insight into astrocyte activities in diseases associated with WMH and CVR, like AD.